# Homœopathy v. Common Sense

**Published:** 1868-09-01

**Authors:** H. O. Hitchcock

**Affiliations:** Kalamazoo


### *CORRESPONDENCE.*

---

### CASE OF PUERPERAL CONVULSIONS—HOMŒOPATHY *vs.* COMMON-SENSE.

Mr. Editor,—Sure that too much light from experience can not be thrown upon so frightful an aberration of the nervous system, as is known under the name of "Puerperal Convulsions," and aware that in the light of such cases, the relative merit of the various "pathies" and common-sense medicine becomes especially evident, I am induced to send to the Journal the following notes.

Mrs. B., a stout young Irish woman, of very full habit, was delivered of her first child on the morning of August 5th, at 4 o'clock. The attending doctor advertises as follows: "*Obstetrics—Specialty.* My patients have always the happy result of short and easy labor, instead of dread and suffering. Mild, pleasant (in homœopathic form) remedies, free. Low fees."

One hour after her delivery, she was seized with a severe convulsion. These continued, with more or less frequency, until 3 P. M. of the same day. A hygieno-homœopath had been called in consultation. At my visit at 3 P. M., she was being convulsed every fifteen to twenty minutes. No urine had been passed through the day, nor for some hours before her delivery; feet and ankles, and, indeed, her whole person, were œdematous. Her convulsions were very strong, and strangulation was imminent from mucus collecting in the air passages. I found the usual two little cups of water +?, and a little one side one sugar pellet that was being carried off by two voracious flies.

I immediately ordered three drachms of *Bromide of Potassium*, *Morphine*, *Chloroform*, and a lancet. I drew off two ounces of fair colored urine; bled her to three pints; gave in four hours two drachms of the *Bromide*, and kept her under the influence of *Chloroform* for three hours and a half; convulsions were not repeated. This, followed by a little diuretic and aperient, and a comfortable night, found her the next morning completely convalescent.

CONTRAST.

| HOMŒOPATHIC SERIES. | COMMON-SENSE SERIES. |
|---|---|
| Two little solutions of ? One sugar pellet, and two flies carrying it off. Convulsions increasing in force and frequency. Death imminent. | Three pints of blood; two drachms *Bromide of Potassium;* three ounces of Squibb's *Chloroform.* Convulsions ceased. Convalesence commenced. |

H. O. HITCHCOCK, M.D.

KALAMAZOO, *August 8th*, 1868.

